# Associations of continuum beliefs with personality disorder stigma: correlational and experimental evidence

**DOI:** 10.1007/s00127-023-02543-8

**Published:** 2023-08-07

**Authors:** Johannes Stricker, Louisa Jakob, Reinhard Pietrowsky

**Affiliations:** Department of Experimental Psychology, Heinrich Heine University Düsseldorf, Universitätsstraße 1, 40225 Düsseldorf, Germany

**Keywords:** Stigma, Continuum beliefs, Personality disorder, Intervention

## Abstract

**Purpose:**

A pervasive and deeply entrenched stigma of personality disorders exists. For other mental disorders, a large body of research suggests that continuum beliefs (i.e., the endorsement of continuum perspectives on mental health and psychopathology) stimulate more favorable attitudes toward affected persons. Additionally, mental disorder classification systems increasingly incorporate continuous personality disorder models. Yet, it is unclear how continuum beliefs are related to personality disorder stigma. This study evaluated the link of continuum beliefs with personality disorder stigma based on correlational and experimental data.

**Methods:**

A large general population sample (*N* = 848) completed self-report measures of continuum beliefs regarding personality disorders, desired social distance, and prejudice toward persons with personality disorders. Additionally, participants were randomly presented with information supporting a continuous or a dichotomous view of personality disorders.

**Results:**

Continuum beliefs were associated with lower desired social distance (*r* = − 0.19) and prejudice (*r* = − 0.22). Additionally, the brief continuum intervention was associated with increased continuum beliefs (*d* = 0.99) and decreased desired social distance (*d* = − 0.14) and prejudice (*d* = − 0.17). Finally, the intervention effects on desired social distance and prejudice were mediated by continuum beliefs.

**Conclusion:**

This study suggests that highlighting continuum views on personality disorders in public communication and interventions might reduce personality disorder stigma.

**Supplementary Information:**

The online version contains supplementary material available at 10.1007/s00127-023-02543-8.

## Introduction

Personality disorders^1^ are among the most severely stigmatized mental disorders [1, 2]. Stigma, in turn, negatively affects the lives of persons with mental disorders (e.g., [3]). Recent meta-analytic evidence suggests that continuum beliefs (i.e., perceiving mental disorders as a continuum from healthy functioning to severe symptomatology) are associated with more positive attitudes toward affected persons [4]. Additionally, there is robust evidence that interventions can strengthen continuum beliefs [4] and some preliminary evidence that these interventions might also contribute to reducing stigmatizing attitudes [4–7].

Conceptually, personality disorders are increasingly understood continuously [8, 9]. For example, the International Classification of Diseases 11th Revision (ICD-11 [10]) highlights the continuous nature of personality disorders, for which stigma reduction has been a central motive [11]. Yet, it is unknown whether continuous conceptions of personality disorders are truly associated with lower stigma. Thus, this study investigates the link between continuum beliefs and personality disorder stigma in a large general population sample using correlational and experimental methods.

### Personality disorder stigma

Personality disorders are severely stigmatized. For example, common labels associated with persons experiencing personality disorders include “purposefully misbehaving” [12],” manipulative”, or “dangerous” [13, 14]. Also, mental healthcare providers hold more negative attitudes toward persons with personality disorders than toward persons with other mental disorders (e.g., [15–17]). The resulting fear of stigmatization in affected persons may lead to non-disclosure of personality disorder symptoms, which constitutes a treatment barrier (see [19]). Making matters even worse, individuals who exhibit the most pronounced symptoms of personality disorders also report experiencing the most severe stigma [20]. Against this backdrop, the adoption of continuous (or dimensional) conceptualizations of personality disorders has been discussed as a promising approach to mitigating stigma (e.g., [11]).

### Continuous conceptualizations of personality disorders

Personality disorders are characterized by impairments in intrapersonal (e.g., identity) and interpersonal functioning (e.g., interpersonal conflict [10, 21]). Ample research suggests that these impairments lie on a continuum, ranging from adaptive personality functioning to severe dysfunctioning (e.g., [22–24]). This perspective has been adopted in current personality disorder classification systems (e.g., [10]). Consequently, continuous personality disorder conceptualizations will increasingly shape clinical practice and public perceptions of personality disorders. However, thus far, it is unclear whether the shift toward continuous personality disorder conceptualizations may reduce personality disorder stigma.

### Continuum beliefs and mental disorder stigma

Differentiating between “us” and “them” is a central tenet of contemporary stigma models (e.g., [25]). Dichotomous mental disorder conceptualizations imply a supposed differentiation between allegedly “normal” and”disordered” persons. In contrast, continuum models inherently highlight similarities between one’s own experiences and the experiences of persons diagnosed with a mental disorder. Correspondingly, strong empirical evidence indicates that continuum beliefs are related to more positive attitudes toward persons with mental disorders (for a meta-analysis, see [4]). In contrast, dichotomous beliefs (“persons with mental disorders are categorically different from others”) are linked to increased stigma [5, 25, 26].

For personality disorders, no study has, thus far, investigated links between continuum beliefs and stigmatizing attitudes. Given the strong evidence for other mental disorders, it appears plausible that continuum beliefs may be linked to lower personality disorder stigma. However, one might also speculate whether labeling a person’s behavior as categorically different from “normal” experiences may reduce stigmatizing attitudes. For example, in a recent vignette study, the description of borderline personality disorder symptoms consistently produced more negative reactions when they were not accompanied (vs. accompanied) by the respective diagnostic label [27]. Additionally, explicit warnings have been raised regarding the potential of continuous conceptualizations of mental disorders to reinforce the perception that these conditions are merely a “moral weakness” that could be easily overcome if affected individuals truly wanted to (e.g., [28]). Thus, clarification of the link between continuum beliefs and stigma is needed to inform communication about personality disorders. Beyond the correlational level, it is also crucial to determine whether continuum beliefs about personality disorders can be influenced by interventions.

### Continuum interventions targeting mental disorder stigma

Public communication aiming to reduce mental disorder stigma has, historically, used two main strategies [29]: The first strategy (*medicalization*) revolves around categorical beliefs and the second strategy (*normalization*) around continuum beliefs. Interventions providing continuum information reliably increase continuum beliefs. However, continuum interventions are differentially effective for improving stigmatizing attitudes toward persons with mental disorders. Whereas some interventions produced only small and insignificant effects [26, 30, 31], others substantially improved self-reported stigmatizing attitudes. For example, in one study, a group that read a bogus newspaper article supporting continuous conceptions of schizophrenia or depression reported higher social acceptance toward affected persons than groups that received dichotomous or no information [6]. Continuum interventions addressing personality disorder stigma have been explicitly called for [20] but are, thus far, non-existent.

### The present study

This study sought to clarify the correlation between continuum beliefs and personality disorder stigma using a large general population sample. Additionally, we experimentally evaluated the link between continuum beliefs and personality disorder stigma by using a brief continuum (vs. dichotomous) beliefs intervention. First, we hypothesized that continuum beliefs are associated with lower stigma (desired social distance and prejudice) toward tpersons with personality disorders (Hypothesis 1). Second, we hypothesized that presenting participants with information supporting a continuous view of personality disorder leads to higher continuum beliefs than presenting information supporting a dichotomous view (Hypothesis 2). Third, we hypothesized that participants who received continuum information report lower stigma (desired social distance and prejudice) than those presented with dichotomous information on personality disorders (Hypothesis 3). Fourth, we hypothesized that continuum beliefs mediate the intervention effects on stigma (social distance and prejudice; Hypothesis 4).

## Method

### Procedure

We recruited German-speaking adults through different social media/internet channels and flyers. Social media/internet channels included student and regional *Facebook* groups, the website of a popular German psychology magazine, student mailing lists, different *Discord* channels, and the research group’s *Instagram* channel. The flyers were distributed on campus, in local stores, and in supermarkets. The recruitment material advertised a study on the perception of mental health problems. The only inclusion criteria for participation were being of legal age (i.e., ≥ 18 years of age) and having sufficient proficiency in German. We did not specify any additional exclusion or inclusion criteria. Upon clicking a link or scanning a QR code, participants were presented with an online questionnaire on the platform *Qualtrics*. First, participants provided their demographic details. Next, each participant was randomly assigned to the continuum or dichotomy intervention condition. Depending on their intervention condition, participants were presented with one of two vignettes adapted from [6]. Both vignettes were in the form of a brief newspaper article. Participants in the continuum condition read a vignette highlighting the continuity of personality pathology from adaptive personality functioning to severe personality disorder (214 words). This vignette referenced a fictitious researcher supporting a continuum perspective on personality disorder (e.g., “There is no principal difference between people with and without a personality disorder. Whether a personality disorder is present is rather a question of the degree of manifestation of certain symptoms.”). Participants in the dichotomy condition read a similar vignette (224 words), referencing the same fictitious researcher, who, in this intervention condition, supported a dichotomous view on personality disorder (e.g., “If you look at the core symptoms, there is only normal personality or personality disorder, there are no gray areas.”). Table S1 in the Supplementary Information provides the original wordings and English translations of both vignettes. We instructed the participants to read the respective vignette carefully. Additionally, we set a minimum viewing time of 60 s for the vignettes (average viewing time = 80 s for the continuum vignette, 79 s for the dichotomy vignette). After reading the vignettes, the participants completed measures assessing continuum beliefs regarding personality disorders, the desired social distance from persons with personality disorders, and prejudice toward persons with personality disorders. No symptom description was provided. To screen for careless responding, these questionnaires comprised two control questions (e.g., “To ensure the data quality, please select the rightmost response option for this statement (‘fully agree’)”). Finally, we debriefed the participants (see Table S2). The median duration of the experiment was 7.24 min. Data collection started on January 10th, 2022, and was terminated after a predefined period of one month. Hence, we set a predefined time period for data collection and did not a priori specify a sample size. Post-hoc power analyses (α = 0.05, two-tailed) showed that the statistical power was satisfactory for small, medium, and large correlations (83% for *r* = 0.10, 100% for *r* = 0.30, and* r* = 0.50). All participants provided their informed consent prior to study participation. The ethics committee of the Faculty of Mathematics and Natural Sciences at the Heinrich Heine University Düsseldorf approved the study protocol.

### Participants

Overall, *N* = 879 participants completed this online study. After the data collection, we had to exclude one participant who was below 18 years of age. Additionally, we excluded 30 participants who failed to solve both control questions correctly. Thus, the final sample comprised 848 participants (436 in the continuum and 412 in the dichotomy condition). Participants’ age ranged from 18 to 85 years (*M*_age_ = 42.31, *SD* = 14.09). Most participants indicated to be female (76.89%). Additionally, most participants (64.74%) indicated having prior personal or professional experience with the topic personality disorders. Table 1 displays the demographic details for the overall sample and both intervention groups separately. Both groups did not differ statistically significantly in any demographic characteristic (see Table 1).

**Table 1:** Demographic characteristics

Demographic characteristic	Total sample	Continuum condition	Dichotomy condition	*p*
Age (years), *M (SD)*	42.31 (14.09)	42.30 (13.99)	42.33 (14.21)	0.973
Age group, *n* (%)				0.941
18–25 years	123 (14.50)	65 (14.91)	58 (14.08)	
26–35 years	184 (21.70)	94 (21.56)	90 (21.84)	
36–45 years	188 (22.17)	96 (22.02)	92 (22.33)	
46–55 years	175 (20.64)	92 (21.10)	83 (20.15)	
56–65 years	129 (15.21)	68 (15.60)	61 (14.81)	
66 years or older	44 (5.19)	19 (4.36)	25 (6.07)	
Age not provided	5 (0.59)	2 (0.46)	3 (0.73)	
Gender, *n* (%)				0.095
Female	652 (76.89)	335 (76.83)	317 (76.94)	
Male	180 (21.23)	93 (21.33)	87 (21.12)	
Other	9 (1.06)	7 (1.61)	2 (0.49)	
Not disclosed	7 (0.83)	1 (0.23)	6 (1.46)	
Highest educational degree, *n* (%)				0.685
No formal educational degree	4 (0.47)	2 (0.46)	2 (0.49)	
High school degree	479 (56.49)	248 (56.88)	231 (56.07)	
University or college degree	358 (42.22)	184 (42.20)	174 (42.23)	
Not disclosed	7 (0.83)	2 (0.46)	5 (1.21)	
Occupational status, *n* (%)				0.298
Employed or self-employed	514 (60.61)	258 (59.17)	256 (62.14)	
Students or trainees	138 (16.27)	78 (17.89)	60 (14.56)	
Currently not working (e.g., unemployment, retirement)	188 (22.17)	94 (21.56)	94 (22.82)	
Not disclosed	8 (0.94)	6 (1.38)	2 (0.49)	
Prior personal or professional experience with personality disorders, *n* (%)				0.638
Yes	549 (64.74)	276 (63.30)	273 (66.26)	
No	265 (31.25)	141 (32.34)	124 (30.10)	
Not disclosed	34 (4.01)	19 (4.36)	15 (3.64)	

*N* = 879 for the total sample, 436 for the continuum condition, and 412 for the dichotomy condition. *p* = *p*-value associated with the test of statistically significant differences between the two intervention conditions (*t*-tests for continuous and Chi-square tests for categorical variables)

*M* mean, *SD* standard deviation

### Measures

#### Continuum beliefs

We measured continuum beliefs (i.e., the extent to which the participants perceived personality disorder to be on a continuum with normality) using a three-item scale. These items (“Most of us from time to time show symptoms of personality disorders”, “Normal people can have some of the symptoms of personality disorders”, “Given extreme circumstances, many of us could show signs of personality disorders.”) were translated to German and adapted to personality disorders from the three-item Continuity with Normal Scale [32]. Participants rated all items on a 9-point Likert scale ranging from 1 (“lowest agreement”) to 9 (“highest agreement”). Higher scores on this scale indicate higher continuum beliefs. The Continuity with Normal Scale is a valid and reliable measure of continuum beliefs [33].

#### Desire for social distance

We assessed the desire to maintain social distance from persons with a personality disorder with the German version of the 7-item Social Distance Scale (SDS [34, 35]). The scale items were adapted to refer to personality disorders. The German SDS uses a 5-point Likert scale ranging from 1 (“certainly not”) to 5 (“certainly”). We recoded all items so that larger scores reflect a stronger desire to maintain social distance. Previous studies demonstrate the reliability and validity of the German SDS (e.g., [36]).

#### Prejudice toward persons with personality disorders

We assessed prejudice toward persons with personality disorders with the German short version of the Prejudice toward People with Mental Illness Scale (PPMI-D-K [37]). The PPMI-D-K contains 16 items assessing prejudice and stigmatizing attitudes toward persons with mental disorders, which we adapted to refer to personality disorders (e.g., “Those who have a serious personality disorder should not be allowed to have children.”). Participants rated all items on a 5-point Likert scale ranging from 1 (“do not agree at all”) to 5 (“fully agree”). A higher total score of the adapted PPMI-D-K reflects higher prejudice toward persons with personality disorders. Previous research attests to the reliability and validity of the PPMI (e.g., [37, 38]).

### Statistical analyses

**Fig. 1:**
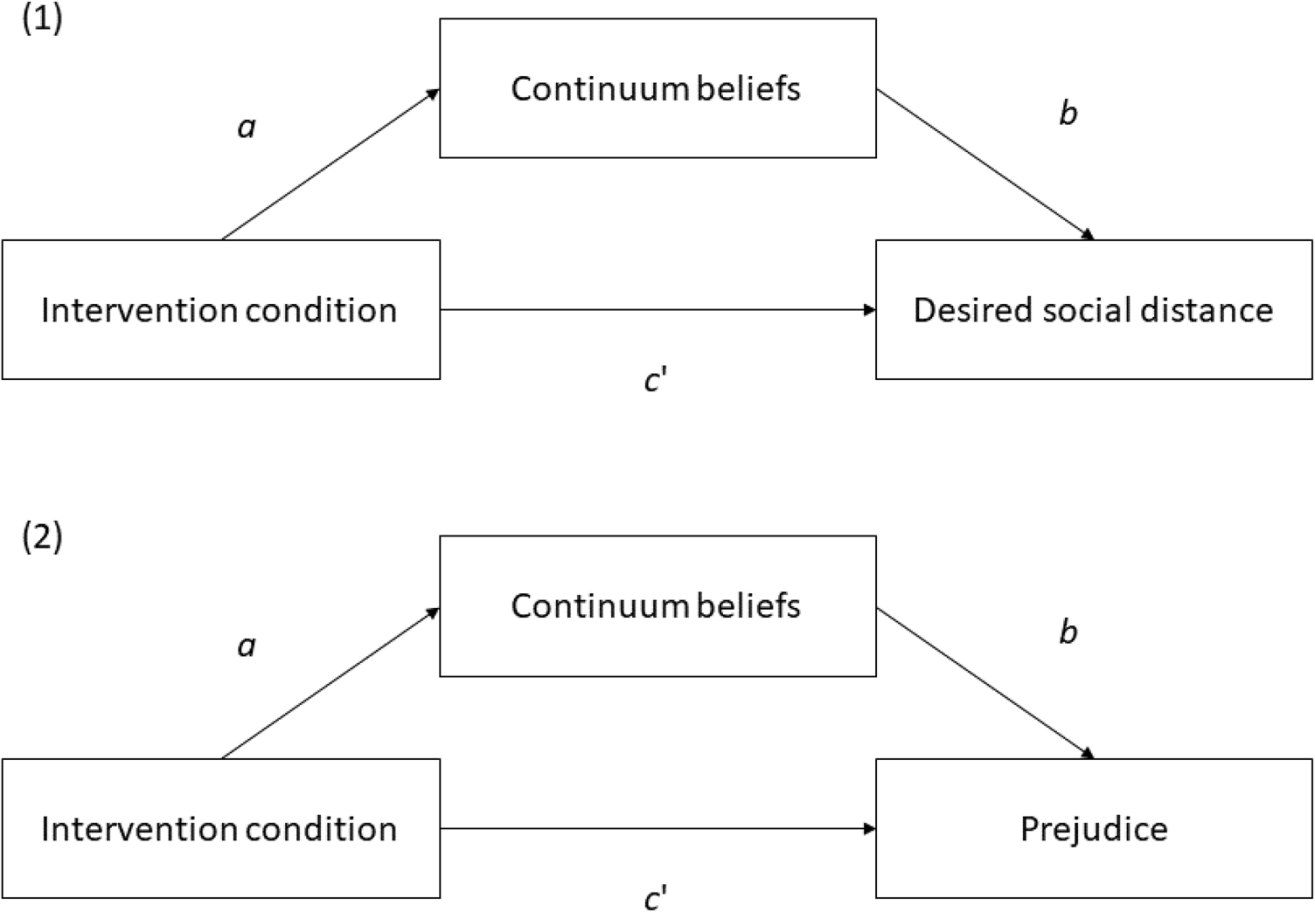
Proposed mediation models depicting the effects of the continuum vs. dichotomy intervention on (1) desired social distance and (2) prejudice toward persons with personality disorders through continuum beliefs

First, we calculated the mean scores and internal consistencies of all measures. Next, we estimated the bivariate correlations of continuum beliefs with the desired social distance and prejudice toward persons with personality disorders. To assess post-intervention between-group differences, we then conducted independent *t*-tests with continuum beliefs, desired social distance, and prejudice toward persons with personality disorders as dependent variables. Finally, we used mediation models to evaluate whether continuum beliefs explain the relations of the intervention condition with desired social distance and prejudice toward persons with personality disorders. Figure 1 displays the proposed mediation models. We used the intervention condition as a binary predictor (0 = continuum, 1 = dichotomy). In both mediation models, path *a* represents the effect of the intervention condition on continuum beliefs. Path *b* represents the effect of continuum beliefs on desired social distance (first mediation model) or prejudice (second mediation model), controlled for the intervention condition. Finally, path *c*’ represents the direct effect of the intervention condition on desired social distance (first mediation model) or prejudice (second mediation model) after controlling for continuum beliefs. The indirect effect of the intervention condition on desired social distance (first mediation model) or prejudice (second mediation model) through continuum beliefs is estimated by calculating the product of paths *a* and *b* (*ab*). If the bootstrapped and bias-corrected 95% confidence interval of *ab* (based on 10,000 iterations) does not include 0, we concluded that continuum beliefs mediated the effect of the intervention condition on desired social distance (first mediation models) or prejudice (second mediation model; see [39]). Additionally, we computed the intervention condition’s total effect (*c*; comprising *c*’ and *ab*) on desired social distance and prejudice*.* We conducted the mediation analyses with the PROCESS 4.1 macro for SPSS, which uses ordinary least squares regression-based path analysis and provides bias-corrected bootstrapped confidence intervals (CI; [39]). As an additional robustness check, we evaluated whether the intervention effects on continuum beliefs, social distance, and prejudice were moderated by age and prior experience with the topic personality disorders using moderated multiple regression analyses in the PROCESS 4.1 macro [39]. All code and data are available via the Open Science Framework: https://osf.io/6vzng/?view_only=a0a717aa0d864391ac3e447f84d034c0.

## Results

### Preliminary analyses

Table 2 displays the means, standard deviations, and internal consistencies of all study variables for the total sample and both intervention groups. All measures displayed satisfactory internal consistency (α = 0.79 to 0.88). The desired social distance and prejudice toward persons with personality disorders correlated positively in the total sample (*r* = 0.67, 95% CI [0.63, 0.70], *p* < 0.001), the continuum group (*r* = 0.66, 95% CI [0.60, 0.71], *p* < 0.001), and the dichotomy group (*r* = 0.67, 95% CI [0.61, 0.72], *p* < 0.001). The Kolmogorov–Smirnov and Shapiro–Wilk tests of normality reached statistical significance for continuum beliefs, desired social distance, and prejudice (all *p*s < 0.001). Thus, as an additional robustness check, we repeated the correlational analyses with Spearman’s (ρ), a non-parametric alternative to Pearson’s *r*. Our bootstrapped mediation analyses are robust to violations of the normality assumption (see[39]).

**Table 2:** Means (M), Standard Deviations (SD), and Internal Consistencies (α) of the Study Variables

Variable	Total Sample			Continuum condition			Dichotomy condition		
	*M*	*SD*	α	*M*	*SD*	α	*M*	*SD*	α
Continuum beliefs	19.38	6.37	0.88	22.12	4.80	0.81	16.48	6.55	0.88
Desired social distance (SDS)	17.73	5.90	0.88	17.33	5.66	0.87	18.14	6.12	0.88
Prejudice strength (PPMI-D-K)	35.88	8.17	0.79	35.22	7.82	0.77	36.58	8.48	0.80

*SDS* Social Distance Scale [34, 35], *PPMI-D-K* adapted German Prejudice toward People with Mental Illness Scale (short version [37])

### Correlations of continuum beliefs with desired social distance and prejudice

Continuum beliefs correlated negatively with desired social distance in the total sample (*r* = − 0.19, 95% CI [− 0.26, − 0.13], *p* < 0.001), the continuum group (*r* = − 0.10, 95% CI [− 0.19, − 0.004], *p* = 0.040), and the dichotomy group (*r* = − 0.24, 95% CI [− 0.33, − 0.15], *p* < 0.001). Additionally, continuum beliefs correlated negatively with prejudice in the total sample (*r* = − 0.22, 95% CI [− 0.29, − 0.16], *p* < 0.001), the continuum group (*r* = − 0.16, 95% CI [− 0.25, − 0.06], *p* < 0.001), and the dichotomy group (*r* = − 0.25, 95% CI [− 0.34, − 0.15], *p* < 0.001). Regarding prior experience with the topic personality disorders, continuum beliefs correlated negatively with desired social distance in persons with prior experience (*r* = − 0.18, 95% CI [− 0.26, − 0.10], *p* < 0.001) and persons without prior experience (*r* = − 0.15, 95% CI [− 0.27, − 0.03], *p* = 0.013). Finally, continuum beliefs correlated negatively with stigma in persons with prior experience (*r* = − 0.18, 95% CI [− 0.26, − 0.10], *p* < 0.001) and persons without prior experience (*r* = − 0.22, 95% CI [− 0.10, − 0.33], *p* < 0.001).

### Intervention effects

Continuum beliefs were more pronounced in the continuum than in the dichotomy condition, *t* (750.91) = 14.24, *p* < 0.001, *d* = 0.99. Additionally, the desired social distance was smaller in the continuum than in the dichotomy condition, *t* (846) = − 1.99, *p* = 0.047, *d* = − 0.14. Finally, persons in the continuum condition reported lower prejudice toward persons with personality disorders than persons in the dichotomy condition, *t* (846) = 2.43, *p* = 0.015, *d* = − 0.17.

### Tests of mediation

In both mediation models, the continuum intervention condition was associated with higher continuum beliefs than the dichotomy intervention condition (Path *a*), *B* = − 5.64, 95% CI [− 6.41, − 4.87], *p* < 0.001. In the first mediation model, continuum beliefs significantly predicted desired social distance after controlling for the intervention condition (Path *b*), *B* = − 0.19, 95% CI [− 0.26, − 0.12], *p* < 0.001). Additionally, allocation to the dichotomy intervention condition was associated with significantly larger desired social distance than allocation to the continuum intervention condition (total effect; *c*), *B* = 0.81, 95% CI [0.01, 1.60], *p* = 0.047). Yet, this was not the case after controlling for continuum beliefs (direct effect; Path *c*’), *B* = − 0.25, 95% CI [− 1.12, 0.62], *p* = 0.570). The indirect effect (*ab*) of the intervention condition on desired social distance via continuum beliefs reached statistical significance, *B* = 1.06, 95% CI [0.58, 1.57], *p* < 0.05.

In the second mediation model, continuum beliefs significantly predicted prejudice toward persons with personality disorders after controlling for the intervention condition (Path *b*), *B* = − 0.30, 95% CI [− 0.39, − 0.20], *p* < 0.001. Additionally, persons in the dichotomy condition reported significantly larger prejudice toward persons with personality disorders than those in continuum intervention (total effect; *c*), *B* = 1.36, 95% CI [0.26, 2.46], *p* = 0.015. The direct effect of the intervention condition (Path *c*’) did not reach statistical significance, *B* = − 0.30, 95% CI [− 1.50, 0.89], *p* = 0.618). Finally, the indirect effect (*ab*) of the intervention condition on prejudice toward persons with personality disorders via continuum beliefs was statistically significant, *B* = 1.66, 95% CI [1.06, 2.31], *p* < 0.05. In sum, the mediation analyses showed that continuum beliefs mediated the intervention effects on desired social distance and prejudice toward persons with personality disorders.

### Robustness checks

Tables S3 to S8 in the Supplementary Information display the complete results of the robustness checks. Age did not moderate the intervention effects on continuum beliefs (*B* = − 0.02, 95% CI [− 0.07, 0.04], *p* = 0.520), desired social distance (*B* = 0.03, 95% CI [− 0.02, 0.08], *p* = 0.261), and prejudice (*B* = 0.02, 95% CI [− 0.06, 0.09], *p* = 0.671). Additionally, prior experience did not moderate the intervention effects on desired social distance (*B* = − 0.39, 95% CI [− 2.11, 1.32], *p* = 0.651) and prejudice (*B* = − 1.68, 95% CI [− 4.04, 0.67], *p* = 0.162). The intervention effects on continuum beliefs were stronger for persons without than with prior experience with personality disorders, *B* = 2.22, 95% CI [0.58, 3.85], *p* = 0.008. However, importantly, allocation to the continuum intervention was significantly associated with higher continuum beliefs in both moderator groups, i.e., persons without (*B* = − 7.20, 95% CI [− 8.55, − 5.86], *p* < 0.001) and persons with prior experience with personality disorders (*B* = − 4.99, 95% CI [− 5.92, − 4.05], *p* < 0.001). Additionally, non-parametric Spearman correlations replicated the negative links of continuum beliefs with desired social distance (ρ = − 0.17, *p* < 0.001) and prejudice (ρ = − 0.21, *p* < 0.001).

## Discussion

Destigmatization of personality disorders is urgently needed (e.g., [40]). For the first time, this study demonstrated that continuum beliefs are associated with lower personality disorder stigma (i.e., desired social distance and prejudice; Hypothesis 1 supported). Additionally, an experimental approach contrasting continuous vs. dichotomous views on personality disorders revealed increased continuum beliefs and decreased personality disorder stigma after highlighting a continuous perspective on personality disorders (Hypotheses 2 and 3 supported). Finally, continuum beliefs mediated the intervention effects on personality disorder stigma (Hypothesis 4 supported).

Our findings add to the literature on continuum beliefs and stigma by demonstrating that continuous disorder conceptions are related to more positive attitudes toward persons with personality disorders. The identified correlations of continuum beliefs with desired social distance and prejudice (*r* = − 0.19 to − 0.22) were highly similar to the previously established meta-analytic correlation of continuum beliefs with desired social distance (*r* = − 0.17) and prejudice dimensions (*r* = − 0.10 to − 0.26) for depression and schizophrenia [4]. Hence, the continuum beliefs-stigma association appears relatively robust across different mental disorders, including personality disorders.

This study’s experimental results supported the correlational findings. The continuum intervention was associated with decreased desired social distance and prejudice, independently of participants’ age and prior experience with personality disorders. Additionally, the continuum intervention was associated with increased continuum beliefs. This effect was moderated by prior experience with the topic personality disorders but ultimately showed in persons with and without prior experience.

Regarding their magnitude, the intervention results mirror findings from previous continuum interventions: Effects on continuum beliefs were more pronounced than effects on stigma-related outcomes (e.g., [21]). Having said that, for both stigma outcomes, significant between-group differences emerged after a brief continuum intervention (compared to a dichotomy intervention). These differences were explained by between-group differences in continuum beliefs. Given the magnitude of effect sizes, fostering continuum interventions alone will not resolve the problem of mental disorder stigma. Yet, future studies in applied contexts could develop more extensive continuum interventions, building on this proof-of-principle study.

### Limitations and future research

This study has some limitations pointing toward crucial future research directions. First, we used a convenience sample drawn from the general population sample. This approach might have biased the sample composition. Thus, future research using large nationally representative samples is needed. Additionally, future research is needed to assess whether continuum beliefs are also related to lower (self-)stigma in persons with personality disorders and mental health professionals. Second, we assessed continuum beliefs and personality disorder stigma after, but unlike prior work [6], not before the continuum intervention. We did not assess the study constructs twice to avoid reactivity effects that could have arisen if the intervention purpose had been too obvious. This study used a large sample with random allocation to the study conditions. Additionally, there were no significant demographic differences between the two intervention groups, indicating that randomization was successful. Yet, future studies that account for baseline levels of stigma and continuum beliefs are needed to evaluate pre-post intervention changes and differential intervention effects depending on participants’ initial attitudes. Third, this study used informative vignettes resembling newspaper articles rather than descriptions of persons with personality disorders. Thus, participants might have thought of different severity levels or stylistic expressions of personality disorders depending on their prior experiences when responding to the study questionnaires. We used this approach to capture participants’ spontaneous associations with the label “personality disorder”. Yet, future studies should use symptom vignettes or more realistic depictions (e.g., audio or video) that resemble real-life encounters with affected persons more closely. These future studies could also experimentally vary the presented symptoms to evaluate stigmatizing attitudes associated with different levels of personality disorder severity. Such research could also evaluate whether stigma and continuum beliefs differ between different personality disorders or mental disorders described as rather ego-syntonic vs. ego-dystonic. Fourth, due to the proof-of-principle approach of this study, participants read a text highlighting a continuous or dichotomous perspective on personality disorders, and no control group without intervention was included. Hence, further studies using passive control groups are needed to verify whether the continuum or the dichotomy vignette drove the intervention effects. Fifth, we used two well-established stigma measures, but other potentially relevant stigma outcomes were omitted. For example, for other mental disorders, continuum beliefs have, in some studies, been associated with increased blame (e.g., [6]). Personality disorders are sometimes blamed on personal moral weakness (see [12]). Thus, future research is needed to test how continuum beliefs are related to blame attributed to persons with personality disorders.

## Conclusion

Taken together, correlational and experimental evidence obtained in this study indicates that continuum beliefs about personality disorders are related to lower stigma. Thus, continuous personality disorder models are not only evidence-based but may also contribute to destigmatization. Consequently, continuum messages might be included in interventions for personality disorder stigma, and dichotomous conceptions could be deemphasized when communicating about personality disorders.

### Supplementary Information

Below is the link to the electronic supplementary material.

Supplementary file1 (PDF 140 KB)

## Data Availability

All code and data are available via the Open Science Framework: https://osf.io/6vzng/?view_only=a0a717aa0d864391ac3e447f84d034c0.

## Footnotes

1 We use the term “personality disorders” which is commonly used by the general public and authoritative mental disorder classifications. A recently suggested, and potentially less stigmatizing term is “interpersonal disorders” [18].
