# Supplementary material for: Associations of continuum beliefs with personality disorder stigma: correlational and experimental evidence
Source: Soc Psychiatry Psychiatr Epidemiol. 2023 Aug 7;59(9):1629–37. doi: 10.1007/s00127-023-02543-8 (PMC11343845; doi:10.1007/s00127-023-02543-8)
Supplement: Supplementary file 1 — Supplementary file1 (PDF 140 KB) [file 127_2023_2543_MOESM1_ESM.pdf]

SUPPLEMENTARY INFORMATION

Associations of Continuum Beliefs with Personality Disorder Stigma: Correlational and Experimental Evidence

Table S1

Vignettes Used in the Intervention

**Continuum vignette**	
German original vignette	English translation
Gibt es überhaupt einen deutlichen Unterschied zwischen “normaler” Persönlichkeit und Persönlichkeitsstörung?	Is there a clear difference between “normal” personality and personality disorder?
Es existiert kein grundsätzlicher Unterschied zwischen Menschen mit und ohne Persönlichkeitsstörung. Ob eine Persönlichkeitsstörung vorliegt, ist vielmehr eine Frage des Ausprägungsgrades bestimmtes Symptome. Eine aktuelle Studie, die in der Fachzeitschrift World Psychiatry veröffentlicht wurde, zeigte, dass fast jeder von uns ab und zu Symptome einer Persönlichkeitsstörung zeigt.	There is no fundamental difference between people with and without a personality disorder. Whether a personality disorder is present is rather a question of the degree of severity of certain symptoms. A recent study published in the journal World Psychiatry showed that almost all of us show symptoms of a personality disorder from time to time.
Dr. Peter Book von der University of Liverpool, der Hauptautor der Studie, sagt:	Dr. Peter Book of the University of Liverpool, the study’s lead author, states,

„Fast jeder hat ab und zu Symptome, die auch bei einer Persönlichkeitsstörung auftreten. Es ist nur die Frage, wie schwer und dauerhaft diese Symptome sind.“ Häufige zwischenmenschliche Konflikte sind beispielsweise ein Symptom, das bei einer Persönlichkeitsstörung auftritt. Dr. Book erklärt hierzu: „Manche Teilnehmer in unserer Studie berichteten, dass sie ständig Konflikte haben, während andere angaben, dass dies nicht so stark oder nur über kürzere Zeiträume zutreffe.“ Erst wenn die sozialen Beziehungen einer Person dauerhaft stark beeinträchtigt sind, würde man von einer Persönlichkeitsstörung sprechen.	“Almost everyone has symptoms from time to time that also occur in a personality disorder. It’s just a question of how severe and persistent those symptoms are.” Frequent interpersonal conflict, for example, is a symptom that occurs in personality disorders. Dr. Book explains, “Some participants in our study reported having conflicts constantly, while others indicated that this applied not so strongly to them or only over for shorter periods of time.” Only when a person’s social relationships are permanently severely impaired would one speak of a personality disorder
Wissenschaftler nennen dies ein "Kontinuum": Niemand ist zu 100% mental “gesund”. „Zwar brauchen wir das Wort "Persönlichkeitsstörung", um definieren zu können, wer eine Behandlung braucht und wer nicht“, sagt Dr. Book. „Aber was das Erfahren bestimmter Symptome angeht, so haben wir alle damit zu tun, es ist nur eine	Scientists call this a “continuum.” No one is 100% mentally “healthy." “It's true that we need the word "personality disorder" to define who needs treatment and who doesn't," Dr. Book says. "But we all experience certain symptoms, it's just a matter of degree. There's no all-or-nothing. It's a continuum."

Frage des Ausprägungsgrads. Es gibt kein Alles-oder-Nichts. Es ist ein Kontinuum."	
**Dichotomy vignette**	
German original vignette	English translation
Wie können wir Persönlichkeitsstörungen von normaler Persönlichkeit unterscheiden?<br><br>Es gibt klare Unterschiede zwischen Menschen mit und ohne Persönlichkeitsstörung. Eine kürzlich in der Fachzeitschrift World Psychiatry veröffentlichte Studie zeigt, dass Menschen, die an einer Persönlichkeitsstörung leiden, Symptome erleben, die sich deutlich von normalen Erfahrungen unterscheiden.<br><br>Dr. Peter Book von der University of Liverpool, der Hauptautor der Studie, sagt: "Menschen mit einer Persönlichkeitsstörung denken und fühlen völlig anders als andere. Was sie erleben, ist weit außerhalb der Erfahrungen gesunder Menschen." Häufige zwischenmenschliche Konflikte sind beispielsweise ein Symptom, das bei Persönlichkeitsstörungen auftritt. Dr. Book	How can personality disorders and normal personality be distinguished?<br><br>There are clear differences between people with and without personality disorders. A recent study published in the journal World Psychiatry shows that people who have a personality disorder experience symptoms that are markedly different from normal experiences.<br><br>Dr. Peter Book of the University of Liverpool, the study's lead author, says, "People with a personality disorder think and feel completely differently from others. What they experience is far outside the experience of healthy people." Frequent interpersonal conflict, for example, is a symptom that occurs in personality disorders. Dr. Book explains, "Such

erklärt hierzu: “Solche Erfahrungen sind wahrhaftig jenseits der Vorstellungskraft gesunder Menschen.” Ein geschulter Interviewer weiß, wie man diese Erfahrungen erfragt. Dr. Book formuliert es so: “Wenn diese abnormalen mentalen Zustände erkannt werden, kann die Diagnose einer Persönlichkeitsstörung mit großer Sicherheit gegeben werden.”	experiences are truly beyond the imagination of healthy people." A trained interviewer knows how to ask about these experiences. As Dr. Book puts it, "When these abnormal mental states are recognized, a diagnosis of personality disorder can be given with a high degree of certainty."
Gemäß Dr. Book geht das Vorhandensein einer Persönlichkeitsstörung immer mit einem Geisteszustand einher, der sich fundamental von dem, was normal ist, unterscheidet. Er sagt: ”Wenn man die Kernsymptome betrachtet, gibt es nur normale Persönlichkeit oder Persönlichkeitsstörung, es gibt keine Grauzonen. Wir müssen diese spezifischen Krankheiten identifizieren, um den Menschen eine spezielle Behandlung zu ermöglichen.” Dies bringt zum Ausdruck, dass gestörte und normale Persönlichkeit zwei unterschiedliche Kategorien sind, von denen erstere mit einer dauerhaften	According to Dr. Book, the presence of a personality disorder is always accompanied by a mental state that is fundamentally different from what is normal. He states, "When you look at the core symptoms, there is only normal personality or personality disorder; there are no gray areas. We need to identify these specific disorders to provide people specific treatment." This expresses that disturbed and normal personality are two distinct categories, the former of which is associated with a permanent impairment in quality of life. In any case, it is noticeable when a person is affected by a personality disorder.

Beeinträchtigung der Lebensqualität einhergeht. In jedem Fall fällt es auf, wenn eine Person von einer Persönlichkeitsstörung betroffen ist.	

Table S2

Debriefing After the Intervention

German original vignette	English translation
Der Artikel, den Sie gelesen haben, sowie der zitierte Wissenschaftler sind fiktiv. Es besteht in der Wissenschaft heute ein großer Konsens darüber, dass Persönlichkeitsstörungen eher als Dimension („Jeder ist mehr oder weniger stark persönlichkeitsgestört“) anstatt als Kategorie („Entweder man hat eine Persönlichkeitsstörung oder man ist „normal“") anzusehen sind.<br><br>Durch Klick auf „weiter“ bestätigen Sie, dass Sie dieses Debriefing gelesen haben.	The article you read and the scientist quoted are fictitious. There is a large consensus in science today that personality disorders are best viewed as a dimension ("Everyone displays symptoms of a personality disorder to a greater or lesser degree") rather than a category ("Either you have a personality disorder or you are 'normal'").<br><br>By clicking on "continue" you confirm that you have read this debriefing.

Table S3

Results From a Moderated Multiple Regression With Intervention Condition and Age as Predictors of Continuum Beliefs

Predictor	*B*	95% CI	*p*
Intervention condition	-4.85	[-7.27, -2.44]	<.001
Age	-0.04	[-0.08, -.0007]	.046
Intervention condition*age	-.02	[-0.07, 0.04]	.520
R^2	.21		<.001

Note. *N* = 843. Intervention condition was coded as 0 = continuum condition and 1 = dichotomy condition. *B* = unstandardized regression coefficient. 95% CI = 95% confidence interval.

Table S4

Results From a Moderated Multiple Regression With Intervention Condition and Age as Predictors of Desired Social Distance

Predictor	*B*	95% CI	*p*
Intervention condition	-0.62	[-3.01, 1.77]	.612
Age	0.10	[.06, .14]	<.001
Intervention condition*age	0.03	[-.02, 0.08]	.261
R^2			<.001

Note. *N* = 843. Intervention condition was coded as 0 = continuum condition and 1 = dichotomy condition. *B* = unstandardized regression coefficient. 95% CI = 95% confidence interval.

Table S5

Results From a Moderated Multiple Regression With Intervention Condition and Age as Predictors of Prejudice

Predictor	*B*	95% CI	*p*
Intervention condition	0.63	[-2.80, 4.07]	.718
Age	0.09	[0.04, 0.15]	.001
Intervention condition*age	0.02	[-0.06, 0.09]	.671
R^2	.04		<.001

Note. *N* = 843. Intervention condition was coded as 0 = continuum condition and 1 = dichotomy condition. *B* = unstandardized regression coefficient. 95% CI = 95% confidence interval.

Table S6

Results From a Moderated Multiple Regression With Intervention Condition and Prior Experience With the Topic Personality Disorders as Predictors of Continuum Beliefs

Predictor	*B*	95% CI	*p*
Intervention condition	-7.20	[-8.55, -5.86]	<.001
Prior experience	1.24	[0.11, 2.37]	.032
Intervention condition*prior experience	2.22	[0.58, 3.85]	.008
R^2	.23		<.001

Note. *N* = 814. Intervention condition was coded as 0 = continuum condition and 1 = dichotomy condition. Prior experience was coded as 0 = no and 1 = yes. *B* = unstandardized regression coefficient. 95% CI = 95% confidence interval.

Table S7

Results From a Moderated Multiple Regression With Intervention Condition and Prior Experience With the Topic Personality Disorders as Predictors of Desired Social Distance

Predictor	*B*	95% CI	*p*
Intervention condition	1.09	[-0.31, 2.50]	.127
Prior experience	-1.84	[-3.03, -0.66]	.002
Intervention condition*prior experience	-0.39	[-2.11, 1.32]	.651
R^2	.03		<.001

Note. *N* = 814. Intervention condition was coded as 0 = continuum condition and 1 = dichotomy condition. Prior experience was coded as 0 = no and 1 = yes. *B* = unstandardized regression coefficient. 95% CI = 95% confidence interval.

Table S8

Results From a Moderated Multiple Regression With Intervention Condition and Prior Experience With the Topic Personality Disorders as Predictors of Prejudice

Predictor	*B*	95% CI	*p*
Intervention condition	2.67	[0.73, 4.61]	.007
Prior experience	-2.73	[-.4.35, -1.10]	.001
Intervention condition*prior experience	-1.68	[-4.04, 0.67]	.162
R^2	.05		<.001

Note. *N* = 814. Intervention condition was coded as 0 = continuum condition and 1 = dichotomy condition. Prior experience was coded as 0 = no and 1 = yes. *B* = unstandardized regression coefficient. 95% CI = 95% confidence interval.
